# Benefits of Iron supplementation for low birth weight infants: A systematic review

**DOI:** 10.1186/1471-2431-12-99

**Published:** 2012-07-16

**Authors:** Hui Long, Jing-Mei Yi, Pei-Li Hu, Zhi-Bin Li, Wei-Ya Qiu, Fang Wang, Sing Zhu

**Affiliations:** 1 Department of Pediatrics, General Hospital of Chinese People’s Liberation Army, #28 Fuxing Road, Haidian District, Beijing, 100853, China

**Keywords:** Anemia, Infant, Iron deficiency, Iron supplementation, Low birth weight

## Abstract

**Background:**

A number of studies have reported on the effects of iron supplementation in low birth weight infants; however, no systematic review of the available evidence has been conducted to date. Hence, we performed a systematic review of the literature to examine the effects of iron supplementation on hematologic iron status, growth, neurodevelopment, and adverse effects in low birth weight/premature infants.

**Methods:**

We searched the Cochrane Library, Medline, and PubMed for articles reporting on the effects of iron supplementation in low weight infants. The following search terms were used: “preterm born infant(s)/children”; “preterm infants”; “prematurely born children” “weight less than 1500 g at birth”; “born prematurely”; “low birth weight infant(s)”; “infants born preterm”; “prematurity”; “small-for-gestational age”; “very small gestational age infants”; “iron supplementation”; “iron intake”; “iron supplements”; “ferric and/or ferrous compounds”; and “ferrous sulphate/fumarate/sulfate”.

**Results:**

A total of 15 studies were identified and included in the systematic review. Supplemental iron was given orally or as an iron-fortified formula in 14/15 studies. The duration of treatment ranged from 1 week to 18 months. Iron supplementation significantly increased hematologic measures of iron status (including hemoglobin, hematocrit, serum ferritin) relative to placebo or over time in most studies. All controlled studies that examined iron-deficiency anemia (IDA)/ID reported a decreased prevalence of IDA/ID with iron supplementation. Dose dependent decreases in the prevalence of IDA/ID were reported in several studies. Of the 5 studies reporting on growth, none found any significant effect on growth-related parameters (length, height, weight, and head circumference). Only 2 studies reported on neurodevelopment; no marked effects were reported. There were no consistently reported adverse effects, including oxidative stress, inhibited nutrient absorption, morbidity, or the requirement for blood transfusion.

**Conclusion:**

The available data suggest that iron supplementation increases the levels of hematologic indicators of iron status and reduces the prevalence of IDA/ID in low birth weight/premature infants. There is insufficient evidence to make a definitive statement regarding the effects of iron supplementation on growth, neurodevelopment, or the occurrence of adverse effects in low birth weight/premature infants.

## Background

Iron is an essential micronutrient that plays a critical role in many cellular functions and processes, including growth and development. As such, having an adequate supply of iron, along with other micronutrients, is thought to be particularly important for infants [1]. Low birth weight infants, including premature infants, are especially susceptible to developing iron deficiency anemia (IDA) because these infants have smaller iron stores at birth and a greater iron requirement concurrent with a rapid increase in the red cell mass than term infants [2-5]. Increased hemolysis, shortened red blood cell lifespan, low circulating erythropoietin levels, blood sampling, and blood loss associated with medical and surgical procedures may all contribute to anemia in low birth weight and premature infants.

The potential consequences of IDA during infancy include impaired development [6-11] and altered longer term neurodevelopment [12-14]. Due to the high risk of IDA, iron supplementation is recommended for low birth weight infants [15]. Whether or not such supplementation is of any benefit to low birth weight infants is unclear. Interestingly, the findings from several systematic reviews / meta-analyses examining the effects of iron supplementation in children suggest that supplementation improves hematologic markers of iron status [16,17], but does not markedly improve growth, or mental or motor development [18-20]. Although a number of studies have reported on the effects of iron supplementation in low birth weight infants, no systematic review of the available evidence has been conducted to date. Hence, we performed a systematic review of the literature, focusing on the effects of iron supplementation in low birth weight / premature infants on hematologic iron status, growth, and neurodevelopment. We also examined potential adverse effects of iron supplementation.

## Methods

### Search Strategy

The Cochrane Library, Medline, and PubMed databases were searched from inception to June 2011. We limited the searches to include only articles written in English using the following terms: “preterm born infant(s) / children”; “preterm infants”; “prematurely born children” “weight less than 1500 g at birth”; “born prematurely”; “low birth weight infant(s)”; “infants born preterm”; “prematurity”; “small-for-gestational age”; “very small gestational age infants”; “iron supplementation”; “iron intake”; “iron supplements”; “ferric and/or ferrous compounds”; and “ferrous sulphate / fumarate / sulfate”. Boolean operators (not, and, or) were also used in succession to narrow and widen the search. To locate unpublished materials and avoid systemic bias, we manually searched the references lists of original and review articles for symposia proceedings, poster presentations, and abstract from major pediatric association meetings.

### Eligibility Criteria

Studies were eligible for inclusion if they involved infants who were of low birth weight (< 2500 g) or premature (gestational age < 35 weeks) and received iron supplementation through the enteral (formula or iron drops) or parenteral routes. Studies involving supplementation of other micronutrients were also eligible for inclusion if the only difference between the treatment and the control groups was iron supplementation.

Studies were excluded if there was no definite length of treatment available, or if supplementation was combined erythropoietin treatment.

The search and inclusion and exclusion criteria were developed by an investigative team, including experts in the field of pediatric development and nutrient research. Our team included a biostatistician who was instrumental in defining the criteria.

### Study Selection

The first step in study selection was the exclusion of duplicates (ie, reports of the same study). The title and abstract of the trials retrieved in the search were scanned and studies that were obviously irrelevant were excluded. The full text of each remaining study was reviewed to establish eligibility and all relevant information and data were extracted.

### Data Extraction and Quality Assessment

Data were extracted by 3 independent reviewers. Each reviewer used a standardized data collection form to increase uniformity and reduce bias. In the case of discrepancy, the reviewers made a consensus decision. The following information / data were extracted from each eligible study: first author; year of publication; journal; eligibility criteria; definition of premature or low-birth weight; number of cases and control; population demographics; hematological data (hemoglobin, hematocrit, serum iron) before iron supplementation; iron supplement dose; duration of iron supplementation; hematological data after iron supplementation; growth status after iron supplementation; and adverse effects of iron supplementation.

The quality of each study identified for inclusion was assessed using the Delphi list for quality assessment of randomized clinical trials [21].

## Results

### Literature Search

**Figure 1:**
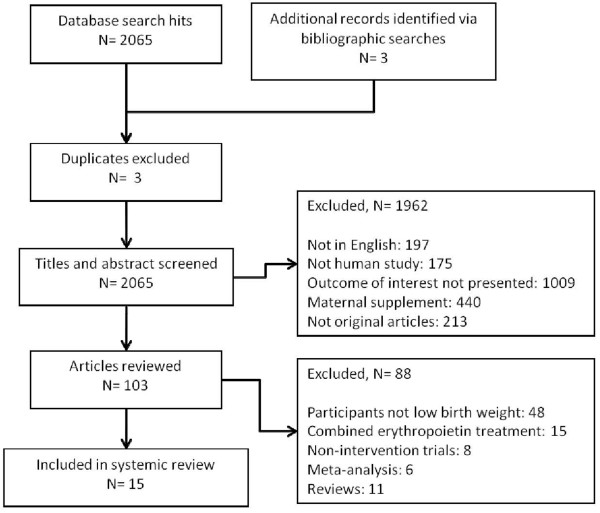
Study flow diagram.

A total of 2065 articles were identified by searching the 3 databases and a further 3 articles were identified by bibliographic searches (Figure 1). Of these articles, 3 duplicates were subsequently excluded. Abstracts and titles for the remaining 2065 articles were reviewed and 1962 were subsequently excluded, typically because outcomes of interest were not presented or maternal supplements were given. Full text review led to exclusion of a further 88 articles. Hence, a total of 15 articles [2,22-35] were included in the systematic review.

### Study Characteristics

The studies included in this systematic review were published between 1960 and 2010 and included low birth weight infants aged from birth to 80 days (Table 1). The number of low birth weight infants in the iron supplementation treatment group ranged from 16 to 90, with most studies having 20 to 40 infants per iron supplementation treatment group. Iron was most commonly given orally [2,23,27,29-35] or as an iron-fortified formula [2,24-26,28]. In 1 study, iron was given by intramuscular injection [22]. The duration of iron supplementation varied considerably, ranging from 1 week up to 18 months.

**Table 1:** Characteristics of the studies examining the effects of iron supplementation in low birth weight infants

**Study**	**Age**^**a**^	**Sample Size**^**b**^	**Eligibility**	**Iron Dose, Duration of** ** Treatment / Follow-up**	**Measurements**
Hammond et al. 1960[22]	2-3 wks	T: 26 C: 22	Premature birth	T: 100 mg intramuscular iron-dextran; C: No iron Treatment: 1 mo; Follow-up: 12 mo	Hematologic: Hb, HCT, PCV, RBC
Brozovic et al. 1974[23]	5 wks	47	GA: 29–37 wks BW: 920–1870 g	36.3 mg/d oral iron Treatment and follow-up: 9 mo	Hematologic: Hb, SI, TIBC
Lundstrom et al. 1977[2]	2 wks	T: 40 C: 50	BW: 1050–2000 g	T: 2 mg/kg/d oral iron; C: No iron Treatment and follow-up: 6 mo	Hematologic: Hb, MCV, reticulocyte count, SI, SF, TRNSAT, TIBC
Iwai et al. 1986[24]	3 mo	Formula: 30 Human milk:15	GA: 30–40 wks BW: 1000–2499 g	Formula: 8 mg/L oral iron Treatment: 3 mo; Follow-up: 6 mo	Hematologic: Hb, RBC, SF, iron, TIBC, MCV
Hall et al. 1993[25]	8-10 d	High iron: 20 Low iron: 23 Human milk: 13	GA: < 35 wks BW: < 1800 g	High: 1.3 mg/kg/d oral iron; Low: 0.3 mg/kg/d oral iron; Milk: 0.3 mg/kg/d iron Treatment: 25–34 d; Follow-up: ~11-13 wks	Hematologic: Hb, MCV, TRNSAT, SF, HCT, RBC, PLFE Development: weight, length, head circumference
Griffin et al. 1999[26]	3 d	A: 29 B: 34 C: 15	GA: ≥ 32 wks BW: < 1750 g	A: 0.9 mg/dL iron formula (1.17 mg/kg/d); B: 0.5 mg/dL iron formula (0.81 mg/kg/d); C: 0.9 mg/dL iron formula until term and then 0.5 mg/dL iron formula (0.86 mg/kg/d) Treatment: 6 mo; Follow-up: 12 mo	Hematologic: Hb, plasma ferritin
Franz et al. 2000[27]	14/61 d	T: 68 C: 65	BW: < 1301 g	T: 2–4 mg/kg/d oral iron once enteral feeding was tolerated; C: No iron Treatment and follow-up: Until 61 days of age	Hematologic: SF, TRNAST, HCT, MCV, MCH, RBC, ID
Friel et al. 2001[28]	Birth	High: 29 Normal: 29	BW: < 2500 g	High: 20.7 mg/L iron formula (0.6-5.9 mg/ kg/d); Normal: 13.4 mg/L iron formula (0.6- 3.0 mg/kg/d) Treatment and follow-up: 12 mo	Hematologic: Hb, HCT, SF, TRN, TRNSAT, MCV, PLFE Development: WTZ, GDA, HTZ Oxidative stress: MDA, PLZN, PLCU, FRAG, CAT, SOD, GHSPx Adverse effects: frequency of infection
Aggarwal et al. 2005[29]	50-80 d	T: 37 C: 36	GA: ≥ 37 wks BW: < 2500 g	T: 3 mg/kg/d oral iron; C: No iron Treatment and follow-up: 8 wks	Hematologic: Hb, SF, microcytic hypochromic, NCHC, NCNC Development: weight, length, head circumference
Miller et al. 2006[30]	7-60 d	T: 16 C: 16	GA: 24–32 wks	T: 3–12 mg/kg/d oral iron; C: No iron Treatment and follow-up: 2–3 wks	Hematologic: RBC, SI, SF, TIBC, ZnPP/H, sTfR Adverse effects: blood and urine isoprostanes
Arnon et al. 2007[31]	2 or 4 wks	2 wks: 32 4 wks: 36	GA: < 32 wks	5 mg/kg/d oral iron Treatment and follow-up: 4–6 wks	Hematologic: SI, SF, sTfR, reticulocyte, Hb Adverse effects: morbidity
Steinmacher et al. 2007[32]	14/61 d	Early: 90 Late: 74	BW: < 1301 g	Early (14 d): 2–4 mg/kg/d oral iron; Late (61 d): 2 mg/kg/d oral iron Treatment: until BW = 1.6x birth BW; Follow up: 5.3 yrs	Neurologic examination, neurophysiological testing (Gross Motor Functioning Classification Scale, Lincoln-Oseretzky Scale, Kaufmann Assessment Battery for Children, visual impairment, and Child Behavior Check List)
Braekke et al. 2007[33]	5 wks	21	GA: < 32 wks BW: < 1500 g	9.4 mg/kg/d oral iron Treatment and follow-up: 1 wk	Hematologic: Hb, reticulocytes, iron, SF, TRNSAT Oxidative stress: urine isoprostane, urine 2,3 dinor, plasma total hydroperoxides Plasma antioxidants: AA, TAA, DHAA, alpha tocopherol, FRAP, GGT, total glutathione
Sankar et al. 2009[34]	2 wks	T: 22, C: 24	BW: < 1500 g	T: 3–4 mg/kg/d oral iron; C: No iron Treatment and follow-up: 60 d	Hematologic: SF, Hb Adverse effects: morbidity
Berglund et al. 2010[35]	6 wks	T1: 78 T2: 82 C: 83	BW: 2000–2500 g	T1: 1 mg/kg/d oral iron; T2: 2 mg/kg/d oral iron; C: No iron Treatment: 6 wks to 6 mo of age; Follow-up: up to 6 mo	Hematologic: Hb, SF, MCV, PLFE, TRN, TRNSAT, sTfR, ID, IDA Development: weight, weight SD score, length, length SD score, head circumference, head circumference SD score, knee-heel length Adverse effects: morbidity

AA, ascorbic acid; BW, birth weight; C, control; CAT, catalase; CGA, corrected gestational age; d, days; DHAA, dehydroascorbic acid; FRAG, red blood cell fragility; GA, gestational age; GDA, Griffiths’ Development Assessment; GHSPx, glutathione peroxidase; GGT, Gamma-glutamyl transferase; Hb, Hemoglobin; HCT, hematocrit; HTZ, height for age z score; MCH, mean corpuscular hemoglobin; MCHC, mean corpuscular hemoglobin concentration; ID, iron deficiency; IDA, iron deficiency anemia; MCV, mean corpuscular volume; MDA, malondialdehyde; mo, month; NA, not available; NCHC, normocytic hypochromic; NCNC, normocytic normochromic; PCV, packed cell volume; PLFE, plasma iron; PLCU, plasma copper; PLZN, plasma zinc; RBC, red blood cell; ROP, retinopathy of prematurity; SF, serum ferritin; SI, serum iron; SOD, superoxide dismutase; sTfR, soluble transferrin receptor; T, iron supplementation group; TAA, total ascorbic acid; TIBC, total iron binding capacity; TRN, transferrin; TRNSAT, transferrin saturation; wks, weeks; WTZ, weight for age z score; ZnPP/H, zinc protoporphyrin to heme ratio.

^a^ Age at the start of supplementation.

^b^ Data analysis sample size.

The quality of studies included in the systematic review was generally acceptable for most items of quality assessment. The majority of studies employed a method of randomization (10/15; 66.7%), had similar between group characteristics at baseline (9/15; 60%), specified eligibility criteria (13/15; 86.7%), provided point estimates and measures of variability for the primary outcome measures (13/15; 86.7%), and included an intention-to-treat analysis (12/15; 80.0%). Only 6/15 (40%) studies provided any definitive information about blinding (outcome assessor, care provider, or patient) (Table 2).

**Table 2:** Quality assessment of studies examining the effects of iron supplementation in low birth weight infants

**Item**	**Was a method of randomization used?**	**Were the groups similar at baseline regarding the most important prognostic indicators?**	**Were the eligibility criteria specified?**	**Was the outcome assessor blinded?**	**Was the care provider blinded?**	**Was the patient blinded?**	**Were point estimates and measures of variability presented for the primary outcome measures?**	**Did the analysis include an intention-to-treat analysis?**
Hammond et al. 1960[22]	Y	Y	NA	NA	NA	NA	Y	Y
Brozovic et al. 1974[23]	NA	NA	Y	NA	NA	NA	Y	N
Lundstrom et al. 1977[2]	N	NA	Y	NA	NA	NA	Y	Y
Iwai et al. 1986[24]	NA	NA	N	NA	N	N	Y	Y
Hall et al. 1993[25]	Y	Y	Y	NA	NA	Y	Y	Y
Griffin et al. 1999[26]	Y	N	Y	NA	NA	Y	N	Y
Franz et al. 2000[27]	Y	Y	Y	NA	NA	NA	Y	N
Friel et al. 2001[28]	Y	Y	Y	NA	NA	NA	Y	Y
Aggarwal et al. 2005[29]	Y	Y	Y	NA	NA	Y	Y	Y
Miller et al. 2006[30]	N	N	Y	NA	NA	NA	N	Y
Arnon et al. 2007[31]	Y	Y	Y	NA	NA	NA	Y	N
Steinmacher et al. 2007[32]	Y	Y	Y	NA	NA	NA	Y	Y
Braekke et al. 2007[33]	N	NA	Y	NA	NA	NA	Y	Y
Sankar et al. 2009[34]	Y	Y	Y	Y	N	Y	Y	Y
Berglund et al. 2010[35]	Y	Y	Y	NA	Y	Y	Y	Y

N, no; NA, information not available or not applicable; Y, yes.

Meta-analyses of the extracted data were not performed because mean values were not available or doses were not adjusted by body weight in the majority of the studies identified. There was significant heterogeneity (data not shown) among the 3 studies in which mean values were available and dose was adjusted for body weight [31,34,35].

### Effect of Iron Supplementation on Hematologic Parameters

All but 1 study reported on the effects of iron supplementation on hematologic measures of iron status (Table 3). The majority of studies reported that iron supplementation significantly increased hematologic measures of iron status relative to control. Specifically, Hammond et al. reported that premature infants treated for 2–4 days with intramuscular iron-dextran (100 mg) had significantly higher hemoglobin concentrations and hematocrit values from 3 months of age onwards and significantly higher erythrocyte counts from 5 months of age onwards compared with premature infants in the control group [22]. In another study, Lundstrom et al. found that low birth weight infants treated for approximately 6 months with an oral iron supplement (2 mg/kg/day) had significantly higher hemoglobin, serum ferritin, mean corpuscular volume (MCV), and transferrin saturation levels at 3 months compared with low birth weight infants in the control group [2]. Iwai et al. similarly reported that low birth weight infants fed an iron-fortified formula (8 mg/L) for 6 months had significantly higher hemoglobin, serum ferritin, and MCV concentrations than control infants fed human milk only [24]. Aggarwal et al. also reported that low birth weight infants fed oral iron in a drop formulation (3 mg/kg/day) for 8 weeks had significantly higher adjusted (for age, hemoglobin, ferritin, and maternal hemoglobin) hemoglobin concentrations from 4 weeks onwards compared with control infants [29]. Finally, Berglund et al. found that marginally low birth weight infants fed an oral iron supplement (1 or 2 mg/kg/day) between 6 weeks and 6 months of age had significantly higher hemoglobin, MCV, ferritin, transferrin, iron, transferrin saturation, and transferrin receptor levels compared with control infants [35].

**Table 3:** Effects of iron supplementation on hematologic parameters in low birth weight infants

**Study**	**Effect**	**No Effect**	**% of ID and/or IDA**	**Conclusion**	**Comments**
Hammond et al. 1960[22]	Hb, HCT significantly higher in T group by 3 mo; erythrocyte count significantly higher in T group by 5 mo	Blood volume, circulating Hb mass	27.3 vs 7.7 % IDA (C vs T)	Early iron suppl. accelerates recovery from early IDA	27 % loss to follow up; other vitamins were administrated; BW and hematologic measurements slightly higher in C vs T group
Brozovic et al 1974[23]	At 3 mo of age, most infants had low serum iron concentrations, which remained low (6–9 mo)	NA	> 50 % IDA	Iron suppl. was insufficient to prevent IDA in most infants	No control group; all infants received vitamin K; some infants received other vitamins
Lundstrom et al. 1977[2]	SF, Hb, MCV, TRNSAT significantly higher in T group by 3 mo	Reticulocyte count	67 vs 0 % ID (C vs T)	LBW infants who do not receive iron suppl. may develop ID by 3 mo of age; 2 mg/kg/d iron is adequate for the prevention of IDA	23 % loss to follow up; after 3 mo of age, an increasing number of C group infants were excluded; SF was higher than normal in C group; iron suppl. given in 2 different forms
Iwai et al. 1986[24]	Hb, SF, MCV significantly higher in the formula group by 4 mo	RBC, SI, TIBC	86 vs 33 % ID (human milk vs formula)	Breast-fed infants have a high risk of ID	Infants in formula group had slightly higher BW than those in human milk group; iron status of LBW infants was not evaluated
Hall et al. 1993[25]	Hospitalization: plasma ferritin lowest in low iron group Discharge: plasma ferritin significantly lower in both formula groups 8 wks after discharge; MCV, MCH significantly lower in low iron group	Hb, HCT, reticulocyte, TRN, TRNSAT	27, 69, 76 % IDA (high, low, human milk)	Preterm infants receive more benefit from receiving preterm infant formula containing 1.3 mg/kg/d iron vs 0.3 mg/kg/d	44 and 25 % of infants in high and low dose groups dropped out because of prematurity related diseases; 13 % of infants in the human milk group completed the study
Griffin et al. 1999[26]	NA	Hb	18 vs 27 % ID (A vs B)	0.81-1.17 mg/kg/d iron seems to meet the iron nutritional needs of preterm infants	In group C, BW was slightly lower, fewer transfusions were received, and Hb was significantly lower vs A and B; no ferritin data for Group C
Franz et al. 2000[27]	NA	All markers of iron nutrition	14.7 vs 40.0 % ID (T vs C)	Fewer infants in group T received blood transfusion vs group C; early iron suppl. is feasible and safe in LBW infants	34 % loss to follow up; group C tended to have > chronic lung disease and ROP
Friel et al. 2001[28]	NA	No difference in any hematologic parameters	6.9 vs 13.8 % ID (normal vs high)	In terms of cognitive outcome, LBW infants did not benefit from high dose iron	No control group
Aggarwal et al. 2005[29]	Adjusted Hb higher in T group	SF	NA	Iron suppl. marginally increases Hb in LBW infants	42 % loss to follow up
Miller et al. 2006[30]	NA	SI, SF, TIBC, sTfR	NA	Corrected reticulocyte count higher in the T group suggesting improved erythropoiesis	Iron suppl. was adjusted by individual iron status; CGA, weight, age at enrollment > in T group
Arnon et al. 2007[31]	Hb, reticulocytes, iron, ferritin significantly higher in the 2 wk group at 8 wks	Reticulocytes, iron, ferritin at 4 wks of age	NA	Iron suppl. to preterm infants as early as 2 weeks of age was more beneficial for iron status, than at 4 weeks of age	All infants given 25 mg/d oral vitamin E; 35 % loss to follow up
Braekke et al. 2007[33]	Iron, TRANSAT significantly increased	Ferritin	NA	Oral iron did not change markers of oxidative stress in LBW infants	No control group; all infants received other vitamins, including vitamin E; 15 % loss to follow up; short length of iron administration
Sankar et al. 2009[34]	NA	SF, HCT, Hb	NA	Iron suppl. at 2 weeks of age did not improve hematological parameters at 2 mo of age in preterm very LBW infants	Iron suppl. group received folic acid and vitamin B12; uncommon iron formulation used
Berglund et al. 2010[35]	All indicators of iron status differed significantly between groups in a dose-dependent manner	NA	2.7, 0, 9.9 % IDA (T1, T2, C) 9.5, 3.8, 35.8 % ID (T1, T2, C)	Marginally LBW infants had higher risk of ID and IDA, especially those exclusively breastfed; 2 mg/kg/d oral iron significantly improved iron status and reduced IDA risk	NA

BW, birth weight; C, control; CGA, corrected gestational age; Hb, Hemoglobin; HCT, hematocrit; ID, iron deficiency; IDA, iron deficiency anemia; LBW, low birth weight; MCV, mean corpuscular volume; mo, month; ROP, retinopathy of prematurity; RBC, red blood cell; SF, serum ferritin; SI, serum iron; sTfR, soluble transferrin receptor; suppl., supplementation; T, iron supplementation group; TIBC, total iron binding capacity; TRN, transferrin; TRNSAT, transferrin saturation; wks, weeks; NA, not available.

In a non-placebo controlled study, Arnon et al. found that premature infants fed enteral iron (5 mg/kg/day) from 2 weeks of age to 8 weeks of age had significantly higher hemoglobin, reticulocyte, iron, and ferritin concentrations compared with premature infants fed enteral iron (5 mg/kg/day) from 4 weeks of age to 8 weeks of age [31]. In another non-placebo controlled study, Braekke et al. reported that very low birth weight infants given oral iron supplementation (9.4 mg/kg/day) from 6 weeks of age had significantly higher iron and transferrin saturation levels at 7 weeks of age [33].

A small number of studies included in the systematic review found that iron supplementation had no significant effect on hematologic measures of iron status relative to control. These include studies by: Friel et al., in which low birth weight infants were fed either a normal (0.6-3.0 mg/kg/day) or high (0.6-5.9 mg/kg/day) iron formula from birth for 12 months [28]; Miller et al., in which 7 to 60 day-old premature infants were given oral iron (3 to 12 mg/kg/day) or control (no iron) for 3 weeks [30]; Sankar et al., in which very low birth weight infants were fed oral iron (3 to 4 mg/kg/day) or control (no iron) from 2 weeks of age until 60 days of age [34].

A study by Griffin et al. compared the effects of formulations containing either 0.9 mg/dL iron or 0.5 mg/dL iron in premature infants and found that there were no dose-dependent differences in hematological indicators of iron status [26]. In another study, Franz et al. reported that early iron supplementation (2 to 4 mg/kg/day once enteral feeding was tolerated) had no significant impact on indicators of iron status in infants with a birth weight <1301 g [27].

### Effect of Iron Supplementation on the Prevalence of Iron Deficiency and Iron-Deficiency Anemia

Most studies reported on the prevalence of iron deficiency (ID) and/or IDA (Table 3). All controlled studies that examined the prevalence of ID and/or IDA found that the prevalence of ID and/or IDA was lower in infants who received iron supplementation compared with infants who did not receive iron supplementation [2,22,24,25,35]. Several studies also reported dose-dependent effects of iron supplementation on the prevalence of ID and/or IDA, with higher iron doses being associated with decreased prevalence [25,26,35]. In contrast, Friel et al. found no obvious effect of dose, with 4/29 (13.8%) infants in the high iron supplementation group and 2/29 (6.9%) infants in the normal iron supplementation group having ID after 12 months of treatment [28]. Franz et al. also found that iron supplementation was associated with a decreased prevalence of ID [27].

Only 1 study found no obvious benefit of iron supplementation on the prevalence of ID and/or IDA. Brozovic et al. reported that oral iron (36.3 mg/day) from 5 weeks to 9 months of age did not prevent ID in premature low birth weight infants [23].

### Effect of Iron Supplementation on Growth and Neurodevelopment

Five studies included in the systematic review examined the effect of iron supplementation on growth, whereas only 2 studies examined the effect of iron supplementation on neurodevelopment (Table 4). None of the studies that examined growth-related variables, including growth rate, length, height, head circumference, and weight, found any effect of iron supplementation [25,28,29,34,35]. With regards to neurodevelopment, Friel et al. found that iron supplementation did not improve cognitive development as measured using Griffiths’ Development Assessment [28]. In the other study, which focused on examining the effects of iron supplementation on neurocognitive development in premature infants weighing < 1301 g, Steinmacher et al. found that early supplementation (< 61 days of age) tended to improve neurocognitive and psychomotor development compared with late supplementation (≥ 61 days of age), as indicated by neurologic examination findings and Gross Motor Function Classification Scores [32].

**Table 4:** Effects of iron supplementation on growth and neurodevelopment in low birth weight infants

**Study**	**Anthropometric Data**	**Neurodevelopmental Outcome**
Hall et al. 1993[25]	No differences in growth rate, length, head circumference	NA
Friel et al. 2001[28]	No differences in WTZ, HTZ at 12 months of age	No difference in GDA; no infant had abnormal development
Steinmacher et al. 2007[32]	NA	More infants in the late iron suppl. group had abnormal neurologic examination; no differences in cognitive development, mobility, hearing, vision, growth; late vs early iron suppl. was not an risk factor for abnormal neurologic examination, disability, or cognitive impairment
Aggarwal et al. 2005[29]	No differences in weight, length, head circumference	NA
Sankar et al. 2009[34]	No difference in weight	NA
Berglund et al. 2010[35]	No differences in weight, length, head circumference	NA

GDA, Griffiths’ Development Assessment; HTZ, height for age z score; suppl., supplement; WTZ, weight for age z score; NA, not available.

### Adverse Effects of Iron Supplementation

Several studies included in the systematic review examined potential adverse effects of iron supplementation, including oxidative stress, inhibition of nutrient absorption, neonatal morbidity, the requirement for blood transfusion, and other adverse effects (Table 5). None of the studies found any evidence that iron supplementation increased oxidative stress in low birth weight infants [28,30,33]. The study conducted by Friel et al. was the only study to examine absorption of other nutrients with iron supplementation and found that both plasma zinc and copper concentrations were significantly higher for the group of infants who received high vs normal iron supplementation [28]. Three of the 5 studies that reported on neonatal morbidity found no differences between the control and treatment groups or dose-dependent effects of iron supplementation on the overall incidence of morbidity [31,34,35]. The other 2 studies found that there was a tendency for there to be a higher prevalence of respiratory tract infection in the iron supplementation group compared with the control group [29] or the high compared with the normal iron supplementation group [28]. Two out of 3 studies examining the need for blood transfusion found no effect of iron supplementation, [30,34] whereas Arnon et al. reported that significantly more infants who started iron supplementation at 4 weeks of age required blood transfusions than infants who started iron supplementation at 2 weeks of age [31]. Another adverse effect reported by Aggarwal et al. included mild vomiting (2/32 infants in the treatment group vs 0/30 infants in the control group) [29].

**Table 5:** Adverse effects of iron supplementation in low birth weight infants

**Study**	**Oxidative stress**	**Inhibition of Other Nutrient Absorption**	**Neonatal Morbidity**	**Blood Transfusion**	**Other Adverse Effects**
Hall et al. 1993[25]	NA	NA	NA	NA	No adverse effects in infants who received higher iron intake
Franz et al. 2000[27]	NA	NA	NA	NA	No adverse effects once enteral feeding (100 mL/ kg/d) tolerated
Friel et al. 2001[28]	No differences in MDA, SOD, CAT between the high and normal groups; GHSPx slightly higher in the high group	PLCU and PLZN significantly lower in high group	Prevalence of respiratory infection greater in the high group	NA	NA
Miller et al. 2006[30]	No differences in blood or urine isoprostanes	NA	NA	No difference	NA
Arnon et al. 2007[31]	NA	NA	No difference	More transfusions in the 4 wk group vs the 2 wk group (10/ 36 vs 1/32)	NA
Braekke et al. 2007[33]	No significant changes in urine isoprostane, 2,3-dinor, total hydroperoxides; plasma antioxidants were largely unchanged	NA	NA	NA	NA
Aggarwal et al. 2005[29]	NA	NA	Prevalence of respiratory infection or bronchiolitis slightly higher in T vs C group (10/32 vs 3/30)	NA	2 infants in the T group reported mild vomiting
Sankar et al. 2009[34]	NA	NA	No difference (19 vs 22 % for T and C groups)	No difference (10 vs 13 for T and C groups)	NA
Berglund et al. 2010[35]	NA	NA	No difference	NA	NA

C, control; CAT, catalase; GHSPx, glutathione peroxidase; MDA, malondialdehyde; PLCU, plasma copper; PLZN, plasma zinc; T, iron supplementation group; SOD, superoxide dismutase; NA, not available.

## Discussion

This is the first systematic review to focus on the effects of iron supplementation in low birth weight / premature infants. Only a relatively small number (N = 15) of studies were identified for review. Several consistent findings were apparent with iron supplementation, including increased concentrations of hematologic indicators of iron status, decreased prevalence of ID / IDA, and a lack of an effect on growth parameters. Few studies that met the criteria for inclusion in our review reported on the effects of iron supplementation on neurodevelopment or the occurrence of adverse effects.

Our observation that iron supplementation generally increased concentrations of hematologic indicators of iron status (notably hemoglobin, hematocrit, and serum ferritin) in low birth weight / premature infants is consistent with the findings of other systematic reviews / meta-analyses which examined the effects of iron supplementation on hematologic indicators of iron status in children [16] and young children [17]. Unsurprisingly, given the improvements in iron status, there were corresponding decreases in the prevalence of ID/IDA among infants who received iron supplementation. Similar to our review, the findings of several systematic reviews / meta-analyses indicate that iron supplementation does not increase growth in children [18,19]. We must emphasize, however, that both the studies included in our review and the previous systematic reviews / meta-analyses typically focused on assessing growth within the first 12 months of supplementation and did not assess longer term growth after iron supplementation. The longer term effect of iron supplementation on growth in low birth weight / premature infants remains to be determined.

As low birth weight / premature infants are particularly vulnerable, and given the fact that humans have no physiologic mechanism for excreting iron, it is important to consider the possibility of iron overload with iron supplementation and identify any potential adverse effects of iron supplementation. Adverse effects of iron supplementation have been suggested, including oxidative stress [36] and an increased risk infection [17]. None of the studies included in our review described any evidence of increased oxidative stress with iron supplementation; however, two studies reported an increased incidence of respiratory tract infection with iron supplementation. Given that iron (and other nutrients) can promote bacterial colonization, an increased risk of respiratory infection is a plausible potential adverse effect of iron supplementation. Further evidence is needed to confirm or deny this possibility. Overall, there were no consistent adverse effects of iron supplementation; however, not all studies monitored for adverse effects of iron supplementation and there was considerable between study variability in the types of adverse effects monitored / reported. Clearly, further studies are on needed on the potential adverse effects of iron supplementation in low birth weight / premature infants.

This systematic review has a number of limitations that must be acknowledged. Perhaps the most important limitation is was the lack homogeneity between studies in terms of treatment dose, length of treatment, timing of treatment, age of the infants, length of follow-up, and variables assessed. These differences clearly make between study comparisons difficult and almost certainly explain some of the variability in findings. Another limitation is that the length of follow-up was relatively short for the assessment of growth and neurodevelopment. It is possible that any beneficial effects of iron supplementation on development may become apparent many years after supplementation has ceased. Indeed, in the only study with follow-up > 18 months, Steinmacher et al. found that more infants who received late vs early iron supplementation had abnormal neurologic examination findings at a median of 5.3 years of age [32]. Although a method of randomization was used in most (10/15) studies included in our systematic review, the number of infants included in all studies was relatively small, thus reducing statistical power. Larger scale studies are clearly needed. With regards to the literature search process, we acknowledge the limitation of only considering English language articles for inclusion.

## Conclusions

In conclusion, our review leads us to suggest that iron supplementation can improve indicators of hematologic iron status and reduce the occurrence of ID / IDA in low birth weight / premature infants. The benefits of iron supplementation on growth appear to be limited, at least in the short term (within 18 months of treatment), whereas there is insufficient evidence on the effects of iron supplementation on neurodevelopment or the occurrence of adverse events in this population. Quite clearly larger scale, randomized controlled trials are needed to determine whether iron supplementation in low birth weight / premature infants affects neurodevelopment (short and long term) and long term growth, and to comprehensively monitor potential adverse effects. Such studies should also aim to determine if specific cohorts of low birth weight / premature infants are more likely to benefit from iron supplementation, and the optimal dose, timing, and duration of iron supplementation.

## Competing interest

We have no competing interests to declare.

## Authors’ contributions

HL screened studies for inclusion, abstracted data, analyzed data, and drafted the manuscript. JY screened studies for inclusion, abstracted data, analyzed data, and drafted the manuscript. PH screened studies for inclusion, abstracted data, analyzed data, and helped to draft the manuscript. ZL verified outcomes data, assessed study quality, analyzed data, and helped to draft the manuscript. WQ, FW, and SZ critically revised the manuscript for important intellectual content. All authors read and approved the final published manuscript.

## Pre-publication history

The pre-publication history for this paper can be accessed here:

http://www.biomedcentral.com/1471-2431/12/99/prepub
